# The complete chloroplast genome of *Craigia yunnanensis*, an endangered plant species with extremely small populations (PSESP) from South China

**DOI:** 10.1080/23802359.2019.1644228

**Published:** 2019-07-23

**Authors:** Hafiz Muhammad Wariss, Yaling Chen, Jing Yang

**Affiliations:** a Yunnan Key Laboratory for Integrative Conservation of Plant Species with Extremely Small Populations, Kunming Institute of Botany, Chinese Academy of Sciences, Kunming, China;; b Key Laboratory for Plant Diversity and Biogeography of East Asia, Kunming Institute of Botany, Chinese Academy of Sciences, Kunming, Yunnan, China;; c University of Chinese Academy of Sciences, Beijing, China

**Keywords:** *Craigia yunnanensis*, Endangered species, plant species with extremely small population (PSESP), plastome, phylogenetic analysis

## Abstract

*Craigia yunnanensis* is an Endangered tree species, enlisted in the national plant species with extremely small population (PSESP) of China for urgent protection. The chloroplast genome is characterized using Illumina pair-end sequencing data. The whole chloroplast genome is 163,166 bp in length and consists of a pair of inverted repeat (IR) regions of 25,573 bp each, which are separated by the large single copy (LSC: 91,580 bp) region and small single copy (SSC: 20,440 bp) regions. The chloroplast genome encodes 113 unique genes, including 79 protein-coding genes (PCGs), 30 tRNA, and 4 rRNA genes. The overall GC content of the whole chloroplast genome is 36.4%. The phylogenetic tree revealed a close relationship between *C. yunnanensis* and *Tilia* species of Malvaceae family.

*Craigia yunnanensis* W. W. Smith & W. E. Evans is a deciduous tree that can grow up to 30 m tall, having a straight trunk and fine wood structure, which produces top-quality timber for construction and furniture (Li 2005; Gao et al. 2010). However, because of deforestation and cash crop cultivation in southern China, its natural population size has been greatly reduced and is on the verge of extinction. As the only surviving species in relic genus *Craigia* (once widespread in the Tertiary era) (Yang et al. 2016), it has been proposed as a second-ranked plant for national protection in China and categorized as ‘Endangered’ by IUCN Red List (Sun 1998; Wang and Xie 2004; IUCN 2008). Moreover, *C. yunnanensis* is now recorded in plant species with extremely small population (PSESP) of China in the national-level Implementation Plan of Rescuing and Conserving China's PSESP (2010e2015). A conservation strategy focusing on PSESP and aimed at rescuing the most endangered plants (Ma et al. 2013). In this study, we characterized the complete chloroplast genome of *C. yunnanensis* to investigate the phylogenetic relationship of *C. yunnanensis* and provide genomic resources for conservation.

Fresh leaves were collected from a wild individuals of *C. yunnanensis* from the Jiangdong town of Dehong prefecture (Yunnan, China; 98°38′E, 24°53′N) and dried using silica gel and voucher specimen (XTBGLJW001-M01) was deposited in Germplasm Bank of Wild Species, Kunming Institute of Botany, Chinese Academy of Sciences. Total genomic DNA was isolated using Tiangen Plant Genomic DNA Kits (Tiangen Biotech, Beijing). Illumina Hi-Seq 2500 were used to generate the 150 bp pair-end reads. There were total 16,626,54 reads for both ends. GetOrganelle pipeline (https://github.com/Kinggerm/GetOrganelle) was used to *de novo* assembling the chloroplast genome, with SPAdes v.3.10.1 as assembler (Bankevich et al. 2012), then visualized by Bandage v.0.8.1 (Wick et al. 2015) to determine paths of the plastome. We used CpGAVAS (Liu et al. 2012) to annotate the chloroplast genome automatically, then manually adjusted and visualized in Geneious Prime v.2019.1.3 (Kearse et al. 2012). A final circular chloroplast genome map was drawn using OGDRAW (Lohse et al. 2013). The newly annotated complete chloroplast genome was submitted to GenBank with accession number MN088379.

The complete chloroplast genome of *C. yunnenensis* is 163,166 bp in length, contains a quadripartite structure that consists of a large single copy (LSC) region of 91,580 bp and a small single copy (SSC) region of 20,440 bp with two inverted repeat (IR) regions of 25,573 bp. It harbors 113 unique genes, including 79 protein-coding genes, 30 tRNA genes, and 4 rRNA genes. The base compositions of *C. yunnanensis* chloroplast genome are uneven (31.4% A, 18.4% C, 18.1%G, 32.2% T), with an overall GC content of 36.4%.

To further examine its phylogenetic position, the maximum likelihood (ML) tree with 1000 bootstrap replicates was inferred by RAxML-HPC2 Workflow on XSEDE (v.8.2.12) CIPRES online portal (Miller et al. 2010) from alignments created by MAFFT (Katoh and Standley 2013) in Geneious Prime v.2019.1.3 (Kearse et al. 2012). We selected 14 published chloroplast genomes including *Aquilaria sinensis* KT148967 and *Aquilaria yunnanensis* MG656407 from Thymelaeaceae and 11 species from Malvaceae, with three species from Brassicaceae as outgroup. The phylogeny reconstructed revealed that *C. yunnanenesis* is closely related to *Tilla* species of Malvaceae (Figure 1). This study will provide a fundamental resource for studying the phylogenetic relationship and conservation of this Endangered plant species with extremely small populations.

**Figure 1.**
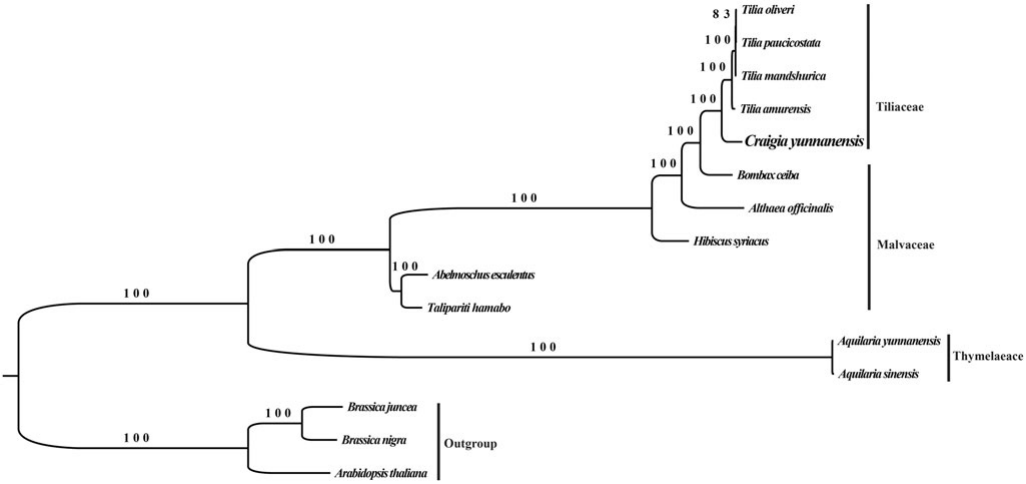
The phylogenetic tree based on 14 complete chloroplast genome sequences. GenBank accession numbers: *Abelmoschus esculentus* (KY635876), *Althaea officinalis* (KY085914), *Aquilaria sinensis* (KT148967), *Aquilaria yunnanensis* (MG656407), *Arabidopsis thaliana* (NC_000932), *Bombax ceiba* (NC_037494), *Brassica juncea* (KT581449), *Brassica nigra* (KT878383), *Craigia yunnanensis* (MN088379), *Hibiscus syriacus* (KP688069), *Talipariti hamabo* (KR259988), *Tilia amurensis* (KT894772), *Tilia mandshurica* (KT894773), *Tilia oliveri* (KT894774), *Tilia paucicostata* (KT894775).
